# Digital Anatomy to Improve Screw Insertion Techniques for Plate-Screw Fixation of the Pubic Body

**DOI:** 10.1155/2018/4690879

**Published:** 2018-12-12

**Authors:** Wenlong Li, Futing Zhao, Zhaoyun Sun, Xing Wang, Shenglong Gao, Bo Xu, Weidong Mu

**Affiliations:** ^1^Department of Orthopedics, Shandong Provincial Hospital Affiliated to Shandong University, 324 Jingwu Weiqi Road, Jinan 250021, Shandong, China; ^2^Department of Orthopedics, Laiwu People's Hospital, 001 Xuehu Avenue, Laiwu 271100, Shandong, China; ^3^Department of Orthopedics, Qingyun County People's Hospital, 777 Qingfeng Road, Dezhou 253000, Shandong, China

## Abstract

**Objectives:**

This study aims to investigate screw insertion sites on the pubic body and the safe screw insertion parameters of a plate-screw fixation system based on the premise of avoiding damage to the inguinal canal and disruption of the rectus abdominis at the pubic symphysis and pubic crest.

**Research Methods:**

Excluding cases with poor image quality, tumors, malformations, and fractures, the data of 80 healthy adults (40 males and 40 females aged from 21 to 83 years old, with an average age of 51.65 years) undergoing a computed tomography (CT) scan of the pelvis between January and June of 2017 were collected from Shandong Provincial Hospital. The CT scans were imported to Mimics® software to reconstruct three-dimensional pelvic models. A 3.5 mm pelvic reconstruction plate was placed starting at the outer edge of the pubic tubercle and along the pelvic brim. The two innermost screw insertion sites were marked. The safe range for the screw insertion sites was then determined. The screw insertion plane was selected to measure the safe screw insertion parameters. The length of the screw, the direction of insertion, and intersex differences were then explored via statistical analyses.

**Results:**

The medial inclination angles (MIAs) of the screw insertion plane for males and females were 30.42±7.95° and 32.88±10.65°, respectively, with no statistically significant differences. For the medial screw, the maximum anterior inclination angle (MAIA), the maximum screw length, and the maximum posterior inclination angle (MPIA) were 46.51±4.01°, 12.40±9.53 mm, and 11.78±10.22° on average, respectively, with no significant differences by gender (P>0.05). For the lateral screw, the MAIA was 10.35±9.46° and showed no gender differences (P>0.05), but the male group had a greater MPIA (male 11.80±11.00° vs. female 6.23±7.91°, P<0.05) and maximum screw length (male 55.71±6.36 mm vs. female 48.68±8.65, P<0.001). For the tangential screw, the maximum screw length, MIA, and anterior/posterior inclination angle (APIA) were 52.19±8.33 mm, 31.65±9.42°, and 7.53±10.18°, respectively, with no significant differences in the angles by gender (both P>0.05), although the screw length in the male group was significantly longer than that in the female group.

**Conclusions:**

Insertion of two screws into the pubic body through a plate from the lateral side of the pubic tubercle is safe and can maintain the origin of the rectus abdominis and the integrity of the inguinal canal compared to traditional plate-screw fixation. Considering that the pubic body is thinner on the lateral side, we suggest a more medial inclination angle for the lateral screw.

## 1. Introduction

The pelvis is a complicated structure comprising the posterior sacrum and bilateral hip bones and exhibits considerable variations in geometrical and mechanical features. Tile et al. [1] found that the posterior ring provides approximately 60% of pelvic stability, whereas the anterior ring provides the other 40%. A finite element analysis of lateral pelvic trauma [2] found that the anterior region of the pelvis is most sensitive to the force of trauma, and the pubic branch was usually fractured first. Although the acetabular anterior column is thinner than the posterior column, the former provides 2.75 times more pelvic stability than the latter [3]. In complicated pelvic and pubic branch fractures, Lei et al. [4] found that fixation of the pubic branch significantly lowers the concentration of stress at the pelvic bone compared with other stabilizing methods, and shifting of the pubic symphysis was reduced. The anterior ring of the pelvis can be fractured easily, and restoring its integrity is critical for improving pelvic stability.

Currently, various fixation methods are available for unstable pelvic outcomes with anterior ring fractures [5–13]. The major treatment methods include external fixation, steel plate-screw fixation (reconstruction and a locking plate), intramedullary screw fixation, and internal fixation via nail insertion. Cases of pelvic and acetabular fractures are increasing as a result of high-energy injuries, and these fractures are typically complicated. Open reduction is inevitable despite disadvantages such as large wounds, high blood loss, and the risk of complications. Maintaining the integrity of the soft tissue structure in the inguinal canal region can significantly reduce the occurrence of iatrogenic inguinal hernia [14]. In clinical practice, we perform minimally invasive internal fixation to treat acetabular anterior column or pubic branch fractures. Two inner screws are placed in the pubic bone through the lateral side of the pubic tubercle at an angle. The inner region of the pubic tubercle is not exposed; therefore, soft tissues (e.g., the rectus abdominis muscle at the pubic symphysis on the inside of the pubic tubercle) are effectively protected. Similarly, open reduction with plate-screw fixation can be performed by inserting a screw at an angle to protect the soft tissues in the inner region. Currently, however, no reference data exist for this modified screw insertion method (i.e., insertion at an angle). Nearby critical tissue structures are at risk of damage if the screw penetrates through the cortex of the bone. In this study, we investigated screw insertion sites and safe insertion parameters based on 3D reconstruction of the normal pelvis using computed tomography (CT) data. This study aims to investigate screw insertion sites on the pubic body and safe screw insertion parameters for a plate-screw fixation system based on the premise of avoiding damage to the inguinal canal and disruption of the origin of the rectus abdominis muscle to improve the safety of screw placement during surgery.

## 2. Materials and Methods

### 2.1. Samples and Equipment

Pelvic X-ray CT data of patients admitted to our hospital for reasons other than fracture between January and June of 2017 were selected. The pelvic structures of the included participants were normal. The 80 adult cases identified included 40 males and 40 females with an average age of 51.65±12.46 years and an age range of 21 to 83 years. None of the patients had anatomical abnormalities or diseases such as pelvic tumors, fracture, malformation, or severe osteoporosis. The included patients had normal pelvic structures and good bone density. The pelvic CT data were saved in digital image and communications in medicine (DICOM) format. The CT scanning parameters were as follows: a 512×512 matrix, 100-140 kV voltage, 360 mA current, 1-mm interslice interval, and 1-1.5 mm slice thickness (SOMATOM Definition Flash, SIEMENS Ltd., USA). The 3D reconstructions using the CT data, screw insertion simulation, and parameter measurements were performed using Mimics 19.0 software (Materialise, Belgium). The hospital ethics committee approved this study under ethical clearance number 2018-102.

### 2.2. Screw Insertion and Data Measurement

#### 2.2.1. Determining the Screw Insertion Sites

The pelvic CT data in DICOM format were imported into Mimics 19.0. Hemipelvis masks were rapidly obtained through CT bone segmentation. These masks were subjected to 3D reconstruction after smoothing.

**Figure 1:**
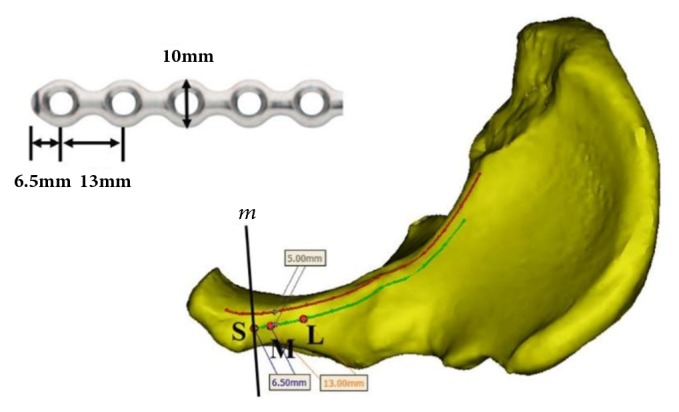
Confirmation of the screw insertion sites. The upper left figure illustrates the measurements of the plate for pelvic reconstruction. The lower right figure displays a top view of the hemipelvis. The red curve illustrates the pelvic boundary. The green curve mirrors the red curve from a distance of 5 mm. The outer edge of the pubic tubercle boundary,* m*, and the green curve intersected at point S, which was the starting point for the plate. The M (6.5 mm away from the S point) and L points (19.5 mm away from the S point) were the insertion sites for the inner and outer screws, respectively.

The commonly used 3.5 mm low-profile reconstruction plate (provided by DePuy Synthes) was measured. The width was 10 mm, the interval between the holes was 13 mm, and the first hole was 6.5 mm from the tip of the plate (Figure 1). The plate was placed starting at the outer edge of the pubic tubercle and along the pelvic boundary to avoid any soft tissue detachment or exposure at the inner pelvis. The insertion sites for the two innermost screws were approximately 5 mm from the pelvic boundary; the insertion sites were 6.5 mm (M point: the inner screw) and 19.5 mm (L point: the outer screw) from the outer pubic tubercle (Figure 1). To better describe these sites, following the direction of the pubic branch, the screw was considered to have an inward inclination if the tip pointed inward or an outward inclination if the tip pointed in the reverse direction. The screw was considered to have an anterior inclination if the tip pointed forward or a posterior inclination if the tip pointed backward.

#### 2.2.2. Obtaining a Safe Region for Screw Insertion

**Figure 2:**
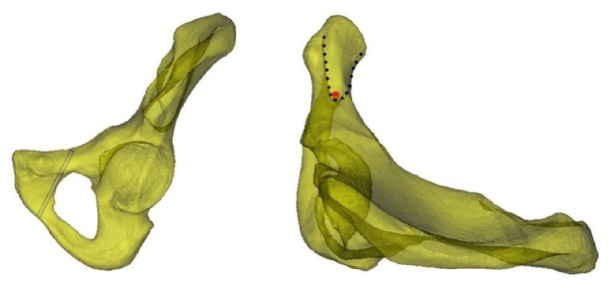
The screw at the line tangential to the obturator. The figure on the left illustrates the simulated screw inserted through the site tangential to the obturator. The safe screw insertion region was the inner region of the screw. The figure on the right illustrates the view of the semitransparent pelvis along the axis of the screw (the red circle is the projection of the screw at the axis). The safe screw insertion region includes the anterior and posterior boundaries that created a V-shape with the tip pointing outward (as indicated by the dotted line).

A 3.5 mm cylinder was employed to simulate the pathway of the screw from the outer screw insertion site, L. The screw was inserted tangential to the inner cortex of the bone to ensure that it remained within the bone. This screw was referred to as the screw at the line tangential to the obturator. When observing from the outside, the screw insertion region was located at the inner pubis (Figure 2, left). When observing the axis of the screw at the tangential line from the top of the semitransparent hemipelvis, the front and back boundaries of the safe screw insertion region were derived (Figure 2, right).

#### 2.2.3. Obtaining the Screw Insertion Plane

**Figure 3:**
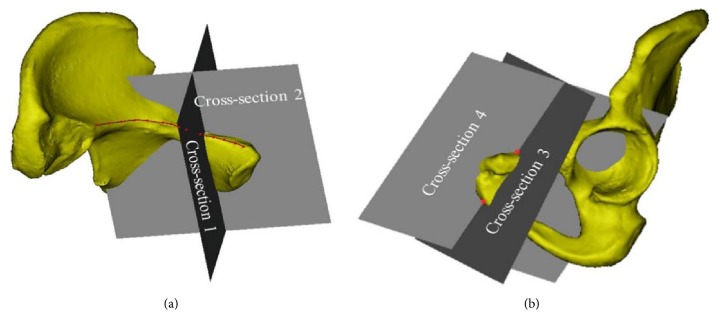
Determination of the cross-sections for screw insertion.

Cross-section 1 was determined using the perpendicular line from point L to the pelvic boundary and the axis of the screw at the line tangential to the obturator. Cross-section 2 was defined by the plane orthogonal to Cross-section 1 and along the axis of the screw at the line tangential to the obturator (Figure 3(a)). Cross-section 3 was defined by the perpendicular line from point M to the pelvic boundary and point O below the pubic symphysis, representing the plane for insertion of the inner screw. Cross-section 4 was orthogonal to Cross-section 3 and passed through points O and M (Figure 3(b)), which is where the internal inclination angle of the screw insertion plane was measured. The insertion plane for the outer screw passed through point L and parallel to Cross-section 3.

#### 2.2.4. Screw Insertion Simulation and Parameter Measurements

**Figure 4:**
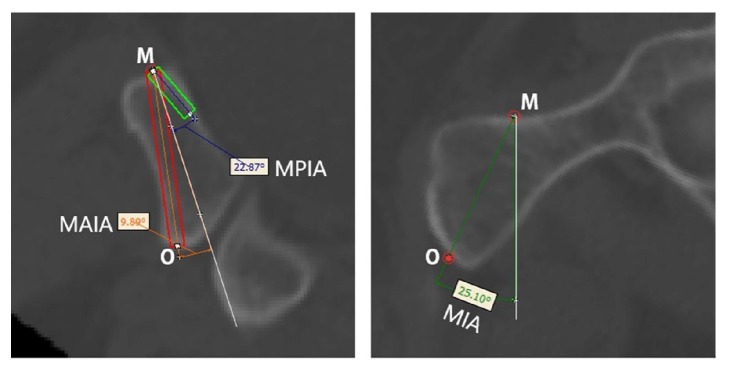
Insertion of the inner screw and parameter measurements. Left: The red rectangle is the screw at the maximum anterior inclination position, whereas the green rectangle is the 14-mm-long screw at the maximum posterior inclination position. The white reference line is perpendicular to the cortical tangential line of screw insertion point M. O indicates the lowest point of the pubic symphysis. MAIA: maximum anterior inclination angle; MPIA: maximum posterior inclination angle. Right: The white reference line is perpendicular to the cortical tangential line of screw insertion point M. The MIA is the medial inclination angle (the angle between line OM and the reference line). O indicates the lowest point of the pubic symphysis.

**Figure 5:**
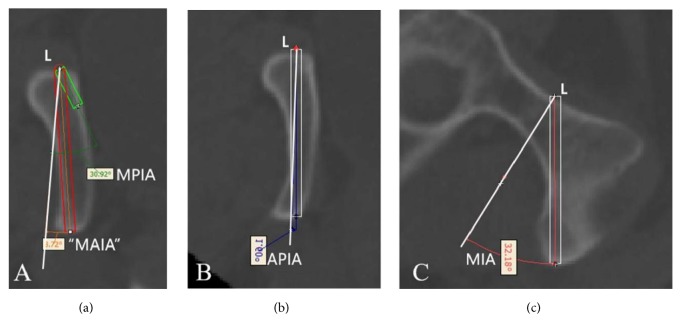
Insertion sites of the outer screw and the screw at the line tangential to the obturator and parameter measurements. (a) Measurement of the parameters of the outer screw. The red rectangle is the screw at the maximum anterior inclination position, whereas the green rectangle is the 14-mm-long screw at the maximum posterior inclination position. The white reference line is perpendicular to the cortical tangential line of the screw insertion point. The MAIA and MPIA are the maximum anterior and posterior inclination angles, respectively. (b) and (c) are Cross-sections 1 and 2, respectively. The white reference line is perpendicular to the cortical tangential line of screw insertion point L. Because the screw at the line tangential to the obturator was fixed, the APIA (anterior/posterior inclination angle) was fixed. When recording the angle, a positive result indicated an anterior inclination angle and a negative result indicated a posterior inclination angle. Figure (c) illustrates the medial inclination angle (MIA). If the angle is less than the MIA, then the screw can penetrate through the obturator.

**Figure 6:**
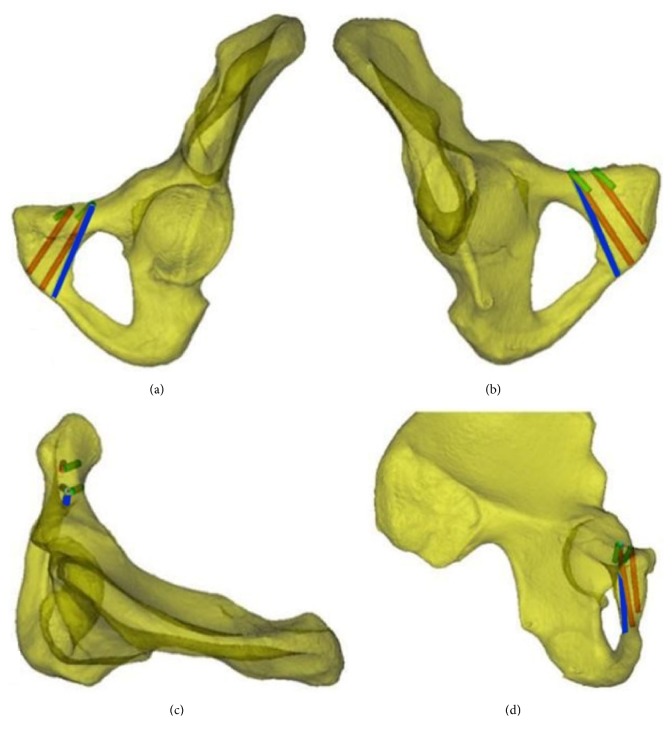
Simulated screw insertion. Figures (a), (b), (c), and (d) display the anterior-outer view, posterior-inner view, top view, and side view, respectively. The red cylinder represents the screw at the maximum anterior inclination position, the green cylinder represents the screw at the maximum posterior inclination position, and the blue cylinder represents the screw at the line tangential to the obturator.

Cylinders with a diameter of 3.5 mm were employed to simulate screw insertion at the aforementioned screw insertion planes. The inner and outer screw insertions were simulated as follows. When leaning forward to the maximum extent, the screw was tangential to the anterior cortex. A 14 mm screw was the shortest. When leaning backward to the maximum extent, the screw was tangential to the posterior cortex. At Cross-section 3, the line passing through point M perpendicular to the cortex was set as the reference line. The angle between the screw and the reference line when maximally leaning forward was the maximum anterior inclination angle (MAIA). The angle between the screw and the reference line when maximally leaning backward was the maximum posterior inclination angle (MPIA) (Figure 4, left). At Cross-section 4, the line passing through point M was considered perpendicular to the cortical tangential line. The angle between this perpendicular line and the OM line was the internal inclination angle. The medial inclination angle (MIA) (Figure 4, right), MAIA, MPIA, and the length of the inner screw, L_1_, at the maximum anterior inclination position of the inner screw were measured. Similarly, the MAIA, MIPA, and length of the outer screw, L_2_, at the maximum anterior inclination position were measured (Figure 5(a)). The MIA and the length of the screw, L_0_, at the line tangential to the obturator were measured (Figures 5(b) and 5(c)). Because the anterior and posterior positions of the screw at the tangential line were fixed, the angle between the axis of the screw and the reference line was referred to as the anterior/posterior inclination angle (APIA). Lastly, the distance between the external boundaries of the pubic tubercles and the distance between the upper and lower boundaries of the pubic symphysis were measured.  Figure 6 illustrates the screw insertion simulation.

Three experimenters who were familiar with Mimics software performed the measurements following the aforementioned steps. The averages of the measured parameters were then subjected to statistical analysis.

## 3. Statistical Analysis

All measurement data were expressed as the mean±standard deviation ($\overset{-}{x}$*±s*) and analyzed using SPSS 19.0. A one-sample Kolmogorov-Smirnov test was employed to ensure that the quantitative data were normally distributed and to examine the homogeneity of variance. Intersex comparisons of the measurement data were performed using two independent-samples t-tests. Differences with* P*<0.05 were considered significant.

## 4. Results

The inner screw insertion site, M, was located 6.5 mm outward toward the pubic tubercle and 5 mm outward toward the boundary. The outer screw insertion site, L, was located 13 mm outward toward the inner screw insertion site, M, and 5 mm outward toward the boundary. From the screw axis at the line tangential to the obturator, the region for screw insertion was defined by the anterior and posterior boundaries and the inner side of the obturator. Because of individual differences, the screw insertion region can exhibit three patterns: a blunt V-shape, a sharp V-shape, and no intersection between the anterior and posterior boundaries.

The MIAs of the inner screw insertion planes were 21.21±4.05° for males and 25.86±8.54° for females, and the intersex difference was significant (*P*<0.05). The MAIA of the inner screw was 12.40±9.53°, L_1_ was 46.51±4.01 mm, and the MPIA was -11.78±10.22° (note that the minus sign indicates posterior inclination both here and in the following sections), with no significant differences in these three parameters between males and females (Table 1). In clinical practice, the insertion angle of the medial screw is suggested to be smaller in males than that in females, but no significant differences were noted in the lengths of the screws used or the forward or backward tilt of the screws. The MAIA of the outer screw was 10.35±9.46°, without an intersex difference. However, the length of the screw for males was significantly longer than that for females (55.71±6.36 mm and 48.68±8.65 mm, respectively). The MPIA was also greater for males than that for females (-11.80±11.00° and -6.23±7.91°, respectively) (Table 2). When inserting a lateral screw, a longer screw can be used, and the backward tilt can be larger for men. The length of the screw at the line tangential to the obturator was 53.16±8.70 mm for males, which was significantly longer than that for females (45.07±10.91 mm;* P*<0.001). No differences in the APIA or MIA were found between the sexes, with values of 7.53±10.18° and 31.65±9.42°, respectively. (Table 3). When inserting a screw at the line tangential to the obturator, a longer screw can be used in men, but the angle does not differ compared to that in women. The distance between the pubic tubercles was greater for females than that for males (61.98±9.04 mm and 58.40±6.03 mm, respectively;* P*<0.05). However, the vertical distance between the pubic tubercles was shorter for females than that for males (39.85±3.26 mm and 41.82±4.65 mm, respectively;* P*<0.05).

Table 1 shows no significant differences between males and females in the safety range between the forward angle and the backward angle or in the maximum lengths of screws.

Table 2 shows differences between males and females for the lateral screws, with longer screws and a larger maximum posterior angle observed in males.

Table 3 refers to the insertion points for the lateral screws, which can be tilted toward the outer side. No differences in angles were observed between males and females, and longer screws were used in males.

## 5. Discussion

The definition of the pubic body differs across the literature. Some define the pubic body as the lower 1/5 of the acetabulum [15], whereas others define it as the flat bone matter in the inner obturator comprising the anterior and posterior sides as well as the pubic symphysis surface [16–18]. The latter definition of the pubic body was applied in the current study. To reduce trauma from open reduction and fixation, we placed the plate starting at the outer edge of the pubic tubercle. The screws were inserted at an angle toward the pubic body to avoid disturbing the pubic soft tissues (e.g., the rectus abdominis muscle at the pubic symphysis, the pyramidal muscle, and the spermatic cord/round ligament). Because the bone matter on the symphysis side of the pubis is thick, whereas the bone matter on the obturator side is thin, an incline is required when inserting screws to avoid penetrating the obturator side and thus minimize the risk of damage to the bladder, obturator nerve, and vessels.

**Figure 7:**
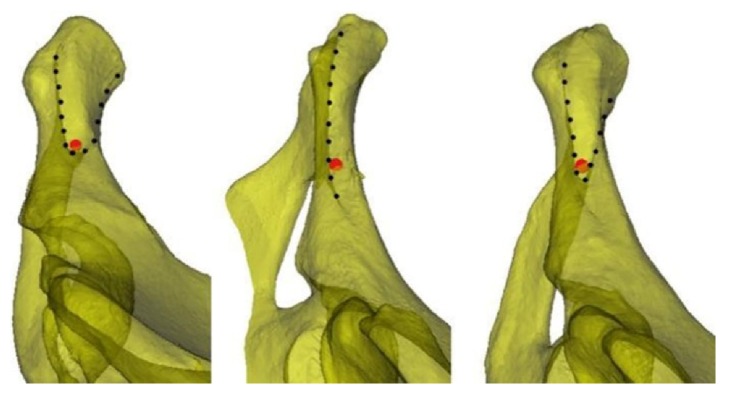
The three patterns of the screw insertion region. Left: The anterior and posterior boundaries show a blunt V-shape. An increase in the outward inclination of the screw at the line tangential to the obturator may result in obturator penetration. Middle: The anterior and posterior boundaries do not intersect. An increase in the outward inclination of the screw at the line tangential to the obturator may result in pelvis penetration. Right: The anterior and posterior boundaries show a sharp V-shape. The screw at the line tangential to the obturator is tangential to the anterior and posterior boundaries. Penetration of the anterior and posterior cortices may occur when the outward inclination of the screw is tangential to the obturator. Note: The red dot in the figure is a simulated axis view of the 3.5-mm screw.

**Figure 8:**
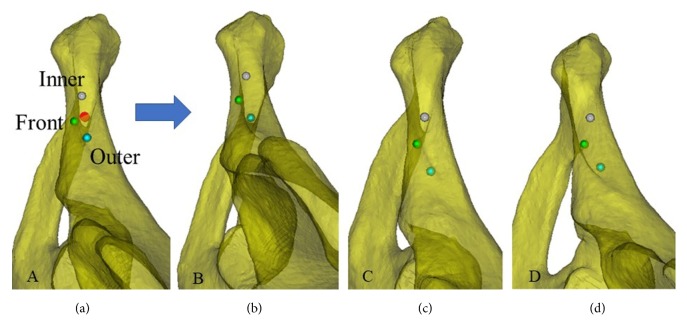
Changes to the screw insertion region for the same pelvis when the screws were inserted at different sites. Three screw insertion sites, front (green), inner (gray), and outer (cyan), are shown at the line tangential to the obturator. When observing the screw insertion region from the axis of the screw at the tangential line, the three aforementioned patterns were observed.

**Figure 9:**
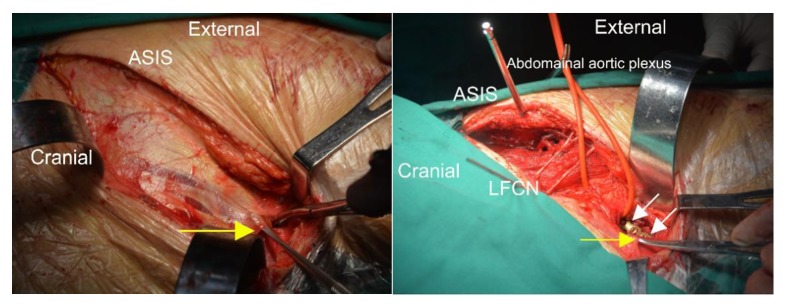
Photos taken during surgery. Left: Exposure of the obliquus externus abdominis and the aponeurosis allows identification of the external inguinal ring (the yellow arrow points to the tissue lifted by the mucosa dissector). Right: The yellow arrow points to the external inguinal ring, and the white arrows point to the two screws set through the plate at the outer edge of the pubic tubercle. The outer edge of the pubic tubercle was exposed by an incision 1 cm from the external inguinal ring. This method effectively avoided exposure of the inguinal canal. Note: ASIS refers to the anterior superior iliac spine, and LFCN refers to the lateral femoral cutaneous nerve.

To avoid treating anterior ring or anterior column fractures by open reduction and fixation and exposing the pubic crest, dissecting the inguinal canal, or cutting the rectus abdominis muscle at the pubic symphysis, this study placed the plate starting at the outer edge of the pubic tubercle and along the pelvic boundary. The insertion sites were determined by the screw holes on the plate. The plate was placed starting at the outer edge of the pubic tubercle along the pelvic boundary. Typically, two screws can be inserted into the pubis from the inner side. Rommens et al. [19] suggested that the recovery after pelvic fracture surgery is strongly correlated with the fracture type and the damage to nearby soft tissues. Because the plate was placed starting at the outer edge of the pubic tubercle, the spermatic cord/round ligament, which starts at the lateral deep surface of the pubic tubercle, passes through the inguinal canal, exits through the external inguinal ring, and passes the medial side of the pubic tubercle, was not compromised. Therefore, an incision in the external inguinal ring and exposure of its contents were not required to provide protection and avoid potential damage (Figure 9). Based on digital anatomy, we found that the improved screw insertion technique is feasible. We also provided a safe screw insertion region, insertion angle, and range of lengths as references for clinical practice. Individual differences exist in the safe insertion region. From the line tangential to the obturator, the anterior and posterior boundaries showed three patterns depending on how they intersected (Figure 7). Further investigation revealed that the insertion boundaries of the corresponding safe region could show three shapes for the same pelvic structure when the positions of the insertion sites changed (Figure 8). Therefore, surgeons should be aware of changes to the safe insertion region.

The average MIA of the inner screw insertion plane was 21.21±4.05° for males and 25.86±8.54° for females. Under the same conditions and with the screw insertion method employed in our study, the distance between the upper and lower boundaries of the pubic symphysis was shorter, and the inner inclination angle was greater when the screw insertion site was more outward (i.e., a greater distance between the pubic tubercles). The measurement results indicated that the distance between the pubic tubercles was greater for females than that for males, but the distance between the upper and lower boundaries of the pubic symphysis was shorter for females than that for males. Therefore, the greater MIA at the screw insertion plane for females may be due to the greater distance between the pubic tubercles and the shorter vertical distance of the pubic symphysis. No significant intersex differences were found for the MAIA, MPIA, or L_1_ of the inner screw. The inner screw can be inserted at an anterior inclination of 12.40±9.53° and a posterior inclination of -11.78±10.22°. The length of the screw can be gradually increased starting from 14 mm at the maximum posterior inclination. At the maximum anterior inclination, the screw length was 46.51±4.01 mm. The MPIA and L_2_ of the outer screw were greater for males than those for females. However, no intersex differences were observed for the MAIA. Therefore, the safe anterior and posterior inclination range is greater for males than that for females. However, compared with the inner screw, the safe inclination range of the outer screw was smaller for both sexes, although the reduced size of the safe range was more significant in females. Regarding the screw at the line tangential to the obturator, except for the greater L_0_ in males, no intersex differences were found for the MIA or APIA. The maximum outward inclination of the outer screw cannot exceed that of the screw at the line tangential to the obturator; otherwise, the nerves and blood vessels at the obturator can be damaged. The screw at the tangential line was at the outermost position, and the direction (i.e., the inward inclination and the anterior/posterior inclination angles) was fixed. A reduction in the inward inclination or an adjustment of the anterior/posterior inclination may result in bone matter penetration and cause potential damage. In clinical practice, one should avoid screw insertion at this location.

Traditional anatomical studies using cadavers are destructive. When multiplane and cross-cutting are required, bones must be dissected into pieces. The operation is difficult and not conducive to research. Errors may result from human factors and affect the accuracy and reliability of study results. In addition, measurement data may be irreproducible. The combination of digital CT data and Mimics software can substantially reduce the resources needed for research, and the corresponding results are reproducible and can be validated with a high level of accuracy. These results can also guide clinical practice [20, 21]. This combination is currently widely applied in fundamental and clinical research [22].

The greatest challenge in digital orthopedics research is image segmentation. Post-image segmentation with 3D reconstruction is the foundation of future research. The precision of image segmentation directly affects the results of follow-up studies and therefore affects clinical practice. This study regarding bone structures was based on CT data. The principle of image segmentation is based on CT values. The higher density of bone provides greater contrast on CT images compared with nearby soft tissues, allowing better differentiation between structures. Therefore, with better image segmentation precision, the reconstructed 3D bone structure is closer to the actual structure. Importantly, however, segmentation of bone versus soft tissue may be worse in cases with severe osteoporosis, and the research results may be adversely affected. With upgraded software, automation, intelligentization, and multifunctional operations can be further advanced. Accordingly, segmentation, reconstruction, meshing, and presurgical design become more realistic, convenient, and efficient.

The measurements in this study have not been performed before, although similar surgical methods are available. Traditionally, the starting point of the rectus abdominis should be dissected and the inguinal canal should be exposed to protect the spermatic cord. What we are exploring is not cutting off the origin of the rectus abdominis. Meanwhile two screws can still be inserted into the pubic body. Our study found that, by placing the plate in this way, the two screws on the medial side of the plate can penetrate into the pubic body, which has a large safety space. We measured the parameters of the safety space and the length of screws, compared the differences between men and women, proved the feasibility of the surgical method, and provided parameters. It provides theoretical support for clinical application.

The shortcoming of this study is the small sample size. More subjects meeting the necessary criteria should be included in our next phase of research. The accuracy and consistency of the measurement data can be further validated using cadavers.

## 6. Conclusions

The insertion of two screws into the pubic body through a plate from the lateral side of the pubic tubercle is safe and can maintain the origin of the rectus abdominis and the integrity of the inguinal canal compared to traditional plate-screw fixation. Considering that the pubic body is thinner on the lateral side, we suggest a more medial inclination angle for the lateral screw. On one hand, penetration into the obturator can be difficult and may result in damage to the obturator nerve and blood vessels. On the other hand, greater screw introversion corresponds to a wider anteroposterior range. We have proved the feasibility of this fixation in this study. Our further study is to study the difference in fixed strength with traditional operation (the two screws are inserted vertically from the upper surface of the pubic body) through biomechanical study.

## Figures and Tables

**Table 1:** Measured parameters for the inner screw ($\overset{-}{x}$*±s*).

Gender	Screw Length, L_1_ (mm)	MAIA (°)	MPIA^#^ (°)
Male (n=40)	47.07±4.08	14.06±8.91	-13.30±10.72
Female (n=40)	45.95±3.93	10.74±9.95	-10.25±9.60
Total (n=80)	46.51±4.01	12.40±9.53	-11.78±10.22
*t *value^∗^	1.261	1.573	-1.341
*P *value^∗^	>0.05	>0.05	>0.05

Note: ^*∗*^*t* and *P* are the results of inter-sex comparisons. ^#^For the MPIA, a negative value indicates that the screw was posterior to the reference line.

**Table 2:** Measured parameters for the outer screw ($\overset{-}{x}$*±s*).

Gender	Screw Length, L_2_ (mm)	MAIA (°)	MPIA^#^ (°)
Male (n=40)	55.71±6.36	9.77±10.53	-11.80±11.00
Female (n=40)	48.68±8.65	10.94±8.33	-6.23±7.91
Total (n=80)	52.19±8.33	10.35±9.46	-9.02±9.92
*t *value^∗^	4.139	-0.553	-2.599
*P *value^∗^	<0.001	>0.05	<0.05

Note: ^*∗*^*t* and *P *are the results of inter-sex comparisons. ^#^For the MPIA, a negative value indicates that the screw was posterior to the reference line.

**Table 3:** Measured parameters for the line tangential to the obturator screw ($\overset{-}{x}$*±s*).

Gender	Screw Length, L_0_ (mm)	MIA (°)	APIA (°)
Male (n=40)	53.16±8.70	30.42±7.95	9.32±9.57
Female (n=40)	45.07±10.91	32.88±10.65	5.74±10.58
Total (n=80)	50.61±11.28	31.65±9.42	7.53±10.18
*t *value^∗^	5.026	-1.167	0.117
*P *value^∗^	<0.001	>0.05	>0.05

Note: ^*∗*^*t* and *P* are the results of inter-sex comparisons.

## Data Availability

The data used to support the findings of this study are available from the corresponding author upon request.
